# Evaluating COVID‐19 Transmission in a Series of Cases and Contacts in Three Municipalities of Colombia: Insights From the WHO First Few X Protocol, August 2020–January 2021

**DOI:** 10.1111/irv.70132

**Published:** 2026-04-07

**Authors:** Damaris Heredia, May Bibiana Osorio, Carolina Figueroa, Susanne Ardila, Dayner Vacca, Magdalena Santos, Daniel Velandia, Marcela Mercado, Maritza González, Franklyn Prieto, Ingrid Garcia, Ángel Rodriguez

**Affiliations:** ^1^ FETP Field Epidemiology Training Program, Public Health Human Talent Training Group National Institute of Health Bogotá Colombia; ^2^ Directorate of Surveillance and Public Health Risk Analysis National Institute of Health Bogotá Colombia; ^3^ Public Health Surveillance Group, Directorate of Public Health Secretariat of Health of Tolima Ibagué Colombia; ^4^ Directorate of Public Health Research National Institute of Health Bogotá Colombia; ^5^ Pan American Health Organization, World Health Organization Washington USA

**Keywords:** contact tracing, COVID‐19, influenza, pandemics, protocol, SARS‐CoV‐2

## Abstract

**Background:**

As part of the framework of preparedness and response to influenza viruses with pandemic potential, the World Health Organization (WHO) has developed a protocol for the characterization of a series of first cases and contacts of an agent with pandemic potential (FFX). At the end of 2019, PAHO/WHO invited Colombia to be among the pilot countries for implementing this protocol. It was conducted by the National Institute of Health of Colombia.

**Methods:**

The WHO generic protocol was reviewed and adapted to Colombia. In its implementation, a series of 99 cases of COVID‐19 and 360 total contacts (159 positive contacts) were evaluated between August 2020 and January 2021 in three municipalities of Colombia. Surveys were conducted, along with RT‐PCR and antibody titer tests. Sociodemographic, clinical, and virological transmission conditions were analyzed to calculate the main epidemiological indicators of viral transmission using measures of central tendency and absolute and relative frequencies.

**Results:**

A total of 258 cases were confirmed to be positive for COVID‐19 for a secondary attack rate of 43%. The most common symptoms were cough, fever, headache, and odynophagia. The percentage of symptomatic contacts, clinical attack rate, incubation period, and serial interval were 34%, 16.2%, 4.6 days, and 4.7 days, respectively.

**Conclusions:**

The significant rate of infection and incubation periods among contacts was similar to those reported worldwide. The implementation of the WHO FFX protocol in a novel context allowed the country to test its capacity to implement global studies to determine early public health interventions. The study faced limitations due to the selection bias towards severe cases and challenges in case recruitment and contact tracing.

## 1 Introduction

At the beginning of a pandemic, there are a lot of unknowns around the epidemiological, clinical, and virological characteristics of the pathogen and its capacity to spread, mainly its human‐to‐human transmission capability and the presence of a population that is partially or totally susceptible to the virus.

Owing to the possible emergence of a new respiratory influenza virus in the world, the Pandemic Influenza Preparedness Program (PIP) [1] was established in 2015 to share information between countries and improve surveillance and response capacity against pandemic risks. Colombia has been among the countries supported by the WHO PIP secretariat since 2017.

Among the tools designed to prepare for and respond to influenza viruses with pandemic potential, the World Health Organization (WHO) developed a protocol for the epidemiological, clinical, and viral characterization of a series of first cases and contacts of an agent with pandemic potential [2, 3, 4] to systematically collect detailed epidemiological and clinical data and establish more precise and effective control measures early.

Colombia participated in the original influenza protocol under the PAHO/WHO initiative at the end of 2019 as part of the PIP activities. However, the implementation of the protocol coincided with the onset of recorded COVID‐19 cases in the country, leading to its adaptation to cover both influenza and SARS‐CoV‐2. This adjustment was carried out by the National Institute of Health of Colombia during the second semester of 2020 and the first quarter of 2021 because of the current response to a pandemic and protocol execution. Technical and financial support from PAHO/WHO was conducted, and the pandemic provided an opportunity to evaluate the protocol in a real‐world context, although its execution required significant efforts in adaptation, training, logistical preparation, and human resources challenges. At the end of July 2020, a total of 286,020 COVID‐19 cases had been confirmed in 38 territorial entities (states). Thus, this project aimed to address existing knowledge gaps by determining the basic epidemiological characteristics of influenza and SARS‐CoV‐2 transmission, particularly in a setting where the early dynamics of the virus were not fully captured. Additionally, it sought to evaluate the protocol's performance in generating strategic data for pandemic response, enhancing ARI surveillance capabilities, and improving public health decision‐making in future emerging infectious threats.

## 2 Methods

A prospective, descriptive, epidemiological study of a case series of people infected with SARS‐CoV‐2 and their contacts was conducted in four hospitals in Colombia in the departments of Tolima, Meta, and Cundinamarca that were selected based on the readiness of surveillance teams, viral circulation conditions, and geographical factors. The sampling followed a *sequential, non‐probabilistic approach*, as typical in First Few X (FFX) investigations, which aim to capture early cases during an outbreak. Cases were included as they sought medical attention at selected hospitals, based on the case definition of the National Institute of Health of Colombia. This method, while suitable for early outbreak characterization, introduces potential *selection biases*. It may overrepresent *moderate to severe cases*, underestimating asymptomatic or mild infections that did not seek care. *Referral bias* could arise from differences in hospital catchment areas, and the sequential nature of case inclusion may introduce *temporal variability* in testing and clinical management. Additionally, *household clustering* may inflate secondary attack rate estimates. Despite these limitations, this approach enabled *rapid real‐time data collection*, critical for guiding early public health interventions^1^ [5]. All confirmed cases were selected until 99 cases were identified from 4 hospitals in 3 departments of Colombia. Typically, an FFX investigation would include the first few confirmed cases in a country; however, this protocol began after the pandemic started between August 2020 and January 2021. It specifically began during the local community transmission phase of the COVID‐19 pandemic and confinement phase of the population to evaluate the protocol performance in a pandemic situation through a pilot test.

The generic protocol developed by WHO was adapted to the epidemiological context and to align with national public health guidelines and operational feasibility. Key adaptations included adjustments in language and terminology to reflect commonly used terms, simplification of demographic data collection for streamlined enrollment and analysis, alignment of case definitions with national health authorities, and modifications to time‐related variables for accurate epidemiological calculations. Six forms were applied on day 1 and day 14, and a symptom diary was given over the phone to confirmed cases and their contacts for 14 days.

The information retrieved from this study was systematically collected according to the formats specified in the protocol; the results were consolidated, tabulated, and analyzed at a global level. This report documents a total of 99 cases and their 360 contacts who were enrolled, evaluated, diagnosed, and monitored as part of the pilot implementation of the “Protocol for the investigation of first cases and their direct contacts (FFX) of pandemic influenza A (HxNy) and novel coronavirus disease 2019 (COVID‐19).”

A descriptive analysis of the sociodemographic, clinical, and virological transmission conditions was performed using measures of central tendency (mean, median) and absolute and relative frequencies (%) to describe the sociodemographic conditions of the population, household characteristics, clinical conditions of cases and contacts, and the main epidemiological indicators of viral transmission: hospitalization rates, proportion of symptomatic individuals, secondary clinical and infectious attack rate, incubation period, serial interval, generation time distribution, case fatality rate, and effective reproductive number (R). Means and medians were calculated for continuous variables such as age, incubation period, and serial interval, while categorical variables such as sex, occupation, and presence of symptoms were summarized using frequency distributions and percentages to facilitate interpretation and comparison across subgroups. Nasopharyngeal swabs and blood samples were collected on days 1 and 14 from symptom onset for laboratory diagnosis. Although additional sampling was not contemplated in the protocol, third and fourth samples were collected for some patients due to operational decisions in the field. The following techniques were used to diagnose the cases:

- *Influenza A virus and SARS‐CoV‐2*: RT‐PCR nucleic acid amplification‐based technique was performed for influenza [6] and *SARS‐CoV‐2* [7].
- *IgG antibodies against SARS‐CoV‐2*: ELISA technique was performed using the ID.VET SARS‐CoV‐2 N IgG indirect test (Innovative Diagnostics, France). The samples from the cases and contacts were processed to detect the presence of anti‐SARS‐CoV‐2 N IgG antibodies [8].
- *Total antibodies:* The WANTAI SARS‐CoV‐2 Ab ELISA kit was used to detect the presence of total antibodies in the cases and contacts having negative IgG results in the first and second serum samples [9].
- *Relative viral load:* To determine the degree of association between viral load and the presence of anti‐SARS‐CoV‐2 IgG antibodies, the viral concentration index values were estimated based on the threshold cycles/number of cycles in which the presence of the virus (Cq) was detected using rRT‐PCR. The retrieved results were grouped in ranges on a logarithmic scale [10].

To analyze the viral transmission conditions, the standard indicators of the original protocol were calculated.

This study was conducted as part of an intensified epidemiological surveillance program in Colombia (emergency response). Studies carried out within the framework of epidemiological surveillance (emergency response) do not require the approval of an Ethics Committee; however, the conditions of confidentiality and ethical considerations for studies in humans defined in Colombian regulations were maintained (resolution 008430 1993). Informed consent was obtained for the collection of information and biological samples from all study participants.

## 3 Results

### 3.1 Study Participants

A total of 99 cases and 360 contacts were recruited for the study between September 2020 and January 2021. They were all living in Colombia, with all participants having a Colombian nationality, except for four contacts who had a Venezuelan nationality.

### 3.2 Sociodemographic Conditions

**FIGURE 1:**
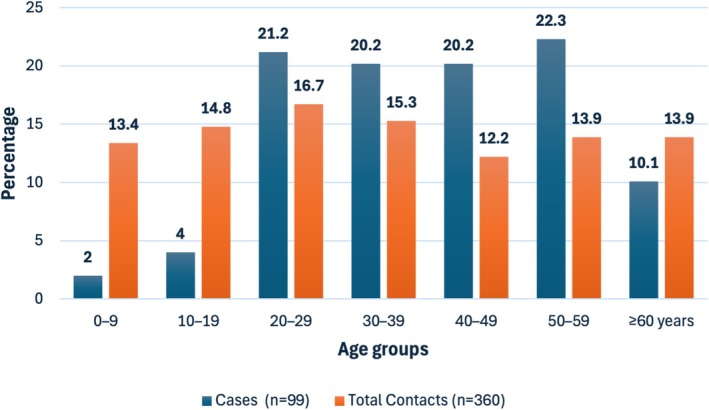
Age distribution of COVID‐19 cases and contacts in Colombia between 2020 and 2021.

The minimum and maximum ages of the cases were 9 and 92 years, respectively, with an average age of 41.3 years. The age of 72.8% of the cases was in the range of 25–54 years. The minimum and maximum ages of the contacts were 7 months and 90 years, respectively, with an average age of 25 years. The age distribution of the contacts was homogeneous in the 5‐year age groups, and the highest number of cases was observed in the group aged ≥ 65 years (35 contacts) (Figure 1).

Of the cases, 21 were health care workers (21.2%) and most of them were nursing assistants, of whom 10 reported occupational exposure. Nineteen contacts were health care workers (5.2%) and most of them were nurses (10 were family members and 9 were co‐workers of the primary case). Two contacts reported symptom onset before the onset of symptoms in the confirmed case (Table 1).

**TABLE 1:** Sociodemographic conditions of the series of cases and contacts with COVID‐19 in Colombia between 2020 and 2021.

Characteristics	Cases		Total contacts	
	*N* = 99		*N* = 360	
	*n*	%	*n*	%
**Sex**				
Male	50	51	159	44.2
Female	49	49	201	55.8
**Occupation**				
Professionals with university degree	23	22.2	31	8.6
Self‐employed	15	15.2	52	14.4
Technician and assistant	14	14.1	15	4.2
Housewife	10	10.1	47	13.1
Unskilled worker	9	9.1	32	8.9
Unemployed/retired	7	7.1	40	11.1
Student	6	6.1	97	26.9
Office worker	4	4	8	2.2
Minor	0	0	26	7.2
Other	11	12	12	3.3
**Health professionals**				
Nurse/nursing assistant	8	8.1	10	2.8
Doctor/emergency doctor	3	3	1	0.3
General services	2	2	0	0
X‐ray technician	2	2	0	0
Administrative assistant	1	1	0	0
Medical coordinator	1	1	0	0
Economist	1	1	0	0
Dentist/oral hygienist	2	2	4	1.1
Occupational health	1	1	0	0
Bacteriologist/laboratory assistant	0	0	3	0.8
Ambulance driver	0	0	1	0.3

Regarding the risk of infection, five people reported attending a mass event, 24.2% of the cases (24) reported being in contact with a person having compatible symptoms, and 21.2% (21) reported being in contact with people having a positive COVID‐19 status.

The households in some cases consisted of 2–5 people, had 2–4 rooms, and had 1–2 people sharing each room (Table 2).

**TABLE 2:** Household characteristics of the series of cases and contacts with COVID‐19 in Colombia between 2020 and 2021.

Characteristics	Cases	
	*N* = 99	
	*n*	%
**Household size/people**		
1	2	2
2	15	15.1
3	26	26.3
4	26	26.3
5	20	20.2
6	6	6.1
7	1	1
8	1	1
9	1	1
SD	1	1
**Number of rooms in the household**		
1	1	1
2	18	18.2
3	42	42.4
4	32	32.3
5	4	4
6	1	1
SD	1	1
**Number of people/rooms**		
1	25	25.2
2	66	66.7
3	5	5.1
4	2	2
SD	1	1
**Age of the household members (years)**		
1–10	51	16.2
11–20	51	16.2
21–30	52	16.7
31–40	44	14.1
41–50	39	12.5
51–60	37	11.8
61–65	19	6.1
71–80	15	4.7
> 80	5	1.6

### 3.3 Clinical Conditions

Ten COVID‐19 cases required hospitalization, including three in intensive care and seven in general wards. Among them, two fatalities were recorded (CFR 20%); both deceased patients were male, aged 52 and 71 years, respectively, with a history of hypertension; one also had obesity as a comorbidity. The primary causes of death were septic shock and multiorgan failure. Additionally, among COVID‐19‐positive contacts recorded, one died—a 66‐year‐old male—with a history of hypertension. According to the death certificate, his cause of death was respiratory failure due to bilateral pneumonia.

**FIGURE 2:**
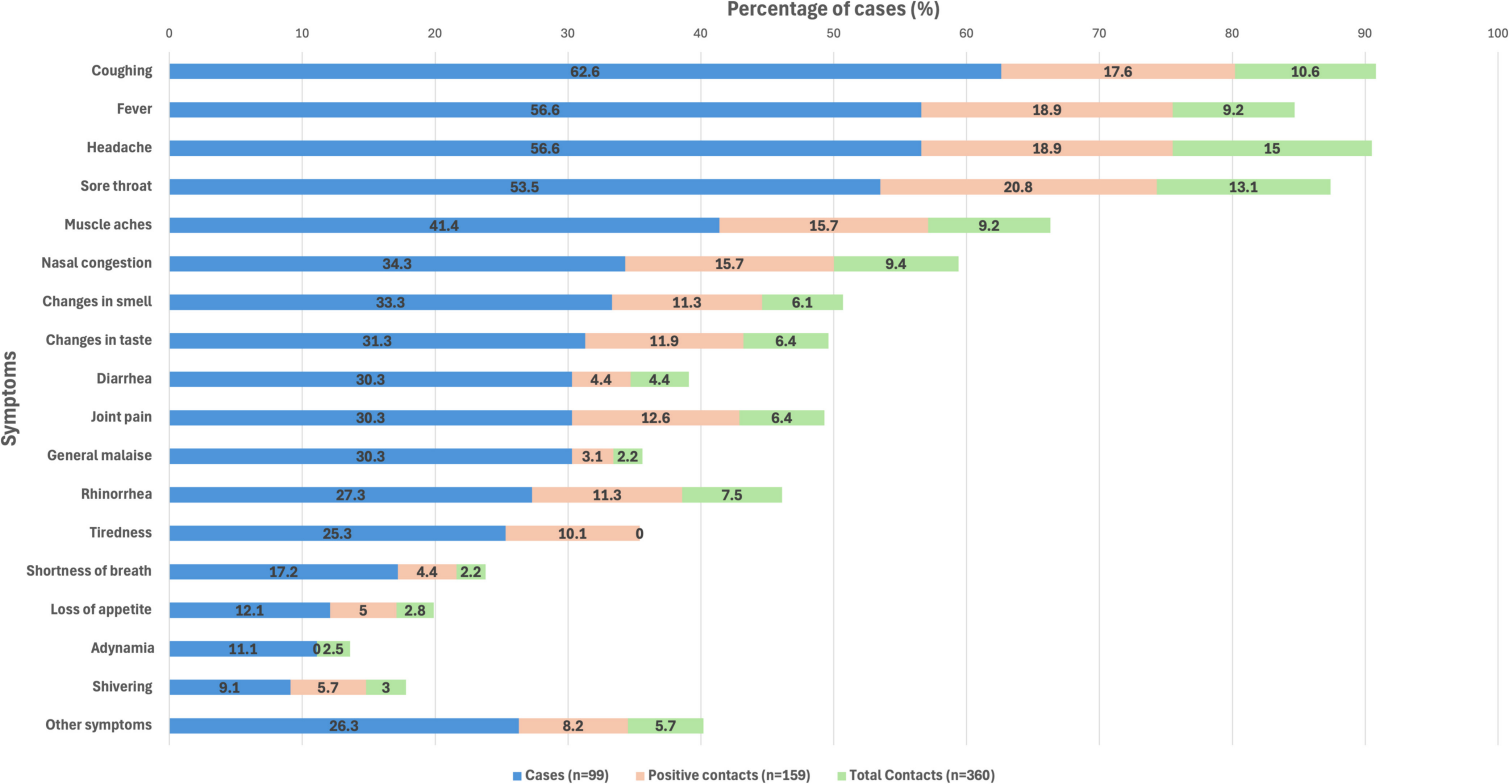
Comparison of symptoms in COVID‐19 cases, positive contacts, and contacts in Colombia between 2020 and 2021.

The most frequent symptoms among the cases were cough, fever, and headache. The most frequent symptoms among the contacts were headache, sore throat, and cough, whereas the most frequent symptoms among the positive contacts were sore throat, fever, and headache (Figure 2). The main pre‐existing health condition in the cases and contacts was hypertension (Table 3).

**TABLE 3:** Comorbidities of the series of cases and contacts with COVID‐19 in Colombia between 2020 and 2021.

Comorbidities	Cases		Total contacts		Positive contacts	
	*N* = 99		*N* = 360		*N* = 159	
	*n*	%	*n*	%	*n*	%
Hypertension	9	9.1	26	7.2	14	8.8
Diabetes mellitus	4	4	10	2.8	4	2.5
Obesity	3	3	6	1.7	3	1.9
Thyroid disease	3	3	5	1.4	2	1.3
Asthma	3	3	7	1.9	3	1.9
Heart disease	3	3	3	0.8	1	0.6
Migraine headaches	1	1	2	0.6	1	0.6
Arthritis	1	1	2	0.6	0	0
Lung disease	1	1	2	0.6	0	0
Other	2	2	5	1.5	3	1.9

### 3.4 Laboratory Results

All cases included in this study had a positive SARS‐CoV‐2 status as confirmed by rRT‐PCR testing, and 8.1% (8 cases) remained positive during follow‐up.

Of the total number of contacts (*n* = 360), 44.2% tested positive for SARS‐CoV‐2 by rRT‐PCR. Among those contacts who tested positive (*n* = 159), 92.5% (*n* = 147) were detected in the first respiratory sample taken, while the remaining 7.5% (*n* = 12) were identified in the second and third respiratory samples taken. Influenza A and B virus‐positive samples were not detected in any cases or contacts who were included in this study.

Ninety‐eight cases and 353 contacts were systematically sampled to assess seroconversion using IgG antibodies, given the necessity of calculating key epidemiological indicators and assessing the transmissibility of the virus. For individuals who tested negative for IgG in all collected samples, total antibodies were analyzed (Table 4). Among the 98 primary cases sampled, 66 demonstrated IgG seroconversion (67%), with 14 cases seroconverting in the first sample (M1) and the remaining 52 in the second sample (M2); the time between symptom onset and the first sample collection ranged from 1 to 17 days, with a median of 4 days.

**TABLE 4:** Samples processed for IgG anti‐N_SARS‐CoV‐2 antibodies and total antibodies for the series of cases and contacts with COVID‐19 in Colombia between 2020 and 2021.

Test	Result	Cases			Total contacts		
		M1	M2	M3	M1	M2	M3
**IgG antibodies**	Inconclusive	2	0		2	1	
	Negative	82	25	1	288	184	7
	Positive	14	62	7	62	94	5
	***Total***	*98*	*87*	*8*	352*	*279*	*12*
**Total antibodies**	Inconclusive	1	1		1		
	Negative	23	16		210	51	
	Positive	5	5		12	7	
	***Total***	*29*	*22*		*223*		

Among the 353 sampled contacts, 112 individuals (31.7%) exhibited seroconversion for IgG, with 60 cases detected in the first measurement (M1) and 52 in the second (M2). An additional 15 contacts (4.2%) demonstrated seroconversion based on the presence of total antibodies, resulting in a cumulative seroconversion rate of 35.9% (127/353). Of these 127 seropositive individuals, 72.4% (92) had previously tested positive by PCR, indicating ongoing viral replication, while 27.5% (35) had negative PCR results, suggesting either a resolved infection or an immune response following asymptomatic exposure. Notably, among the 159 contacts confirmed by PCR, 57.8% (92) exhibited seroconversion, with 80 testing positive for IgG (32 in M1, 48 in M2) and 12 for total antibodies. These findings highlight the variability in immune response timing and reinforce the importance of integrating molecular and serological diagnostics to refine our understanding of SARS‐CoV‐2 transmission dynamics.

Regarding contacts with invalid, negative, or inconclusive PCR results (*n* = 201), 15% (32 contacts) displayed elevated IgG titers, with 28 cases detected in M1 and 4 in M2. Additionally, three contacts tested positive for total antibodies, leading to the identification of seven additional COVID‐19 cases by seroconversion (four IgG‐positive in M2 and three positives for total antibodies). The IgG‐positive cases in M1 under these same conditions (28/353) can be classified as seroprevalent cases (7.9%), suggesting prior exposure rather than active infection.

When laboratory findings were analyzed in relation to symptomatology, 97 out of 166 contacts confirmed by either PCR or seroconversion were asymptomatic (58.4%). This underscores the significant proportion of silent SARS‐CoV‐2 transmission within the studied population, emphasizing the critical role of serological surveillance in capturing the true burden of infection beyond symptomatic cases.

**FIGURE 3:**
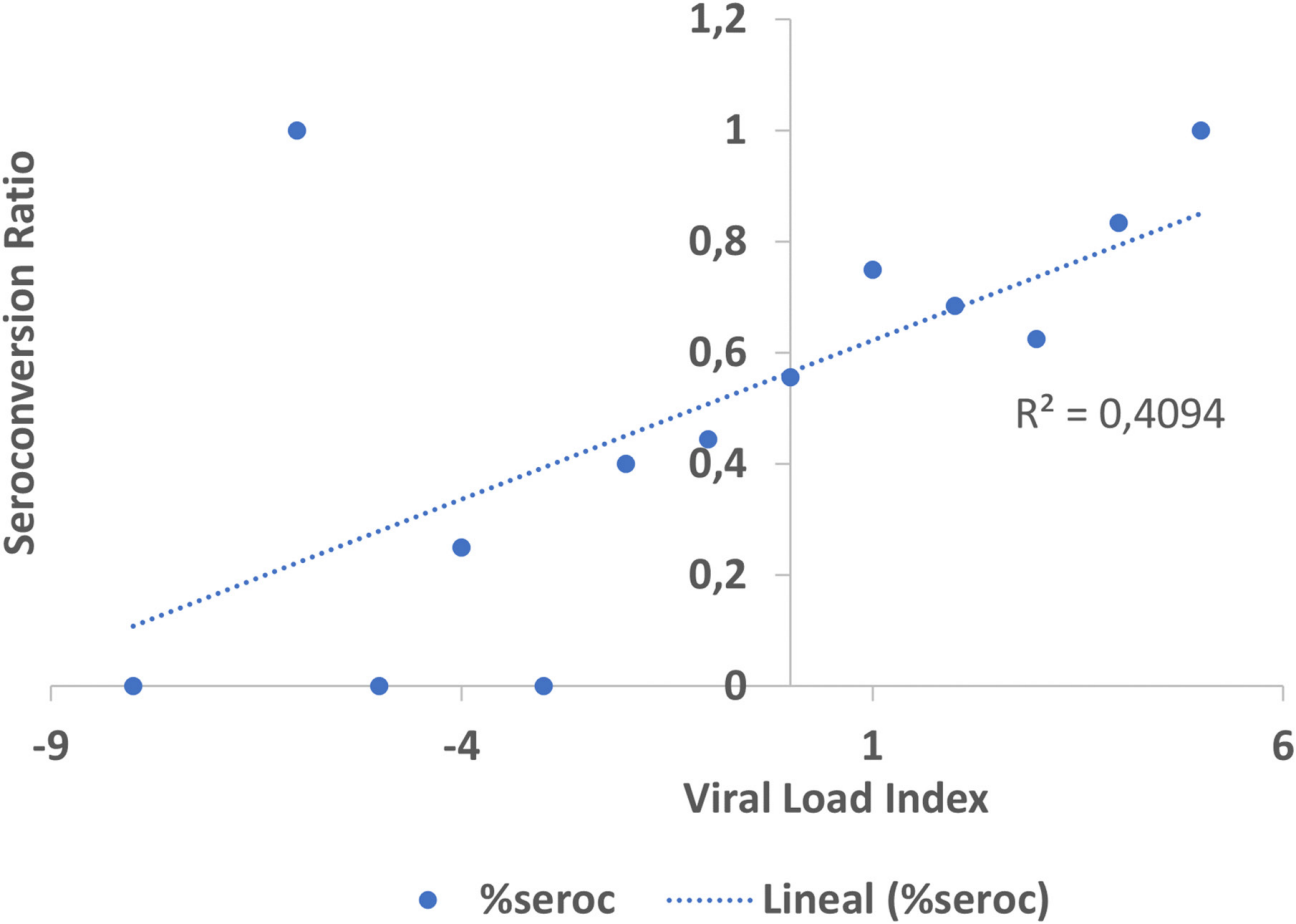
Scatter plot displaying the seroconversion ratio as a function of viral load index (VLI) for the series of cases and contacts with COVID‐19 in Colombia between 2020 and 2021. * The viral load index values are presented as the Log10 of the ratio of the concentrations of viral RNA and human RNA of a constitutively expressed gene (RNaseP).

Patients with a high viral load had a higher proportion of seroconversion than those with a low relative viral load (Figure 3).

### 3.5 Epidemiological Indicators of Virus Transmission

The secondary clinical attack rate was 16.2%, whereas the secondary infection rate was 43%, with an effective reproductive number of 1.47 secondary cases produced by one primary case and a serial interval of 4.7 days (Table 5).

**TABLE 5:** Epidemiological indicators of SARS‐CoV‐2 transmission for the series of cases and contacts with COVID‐19 in Colombia between 2020 and 2021.

Indicator	Sex												Total					
	Female						Male											
	*n*	*N*	%	Aver.	Median	Ratio	*n*	*N*	%	Aver.	Median	Ratio	*n*	*N*	%	Aver.	Median	Ratio
Hospitalization rate	4	132	3				8	126	6.3				12	258**	4.6			
Hospitalization ratio	4	128				0.031:1	8	118				0.067:1	12	246				0.048:1
Proportion of symptomatic	81	132	61.3				86	126	68.2				167	258**	64.7			
Proportion of asymptomatic	51	132	38.6				40	126	31.7				91	258**	35.2			
Secondary clinical attack rate in contacts	27	192	14				28	147	19				55	339*	16.2			
Secondary infectious attack rate in contacts	78	192	40.6				68	147	46.2				146	339*	43			
Incubation period				5	4					4.2	3					4.6	4	
Serial interval				5.8	6					3.6	3.5					4.7	4	
Generation time distribution				5.7	5.5					6.2	6					6	6	
Case fatality rate	0	132				00:01	3	123				0.024:1	3	255				0.011:1
Effective reproductive number (R)	71	43	1.65				60	46	1.3				131	89	1.47			
Lethality percentage	0	132	0				3	126	2.3				3	258	1.16			

## 4 Discussion

This study was conducted in the early stages of the COVID‐19 pandemic, a crucial period for understanding the emergence and spread of new infectious diseases. The findings emphasize the importance of initiating such research promptly, particularly in challenging epidemiological contexts like Colombia. The implementation of the WHO FFX protocol in this novel setting not only tested the country's capacity to execute global surveillance protocols but also proved essential for calculating key indicators of viral transmission. These indicators are critical for determining early public health interventions, enabling a more localized response to contain potential pandemics. The efforts invested in implementing this generic protocol are justified by its value in bridging information gaps, providing early, reliable, and comparable data that can inform global, regional, and country responses to emerging health threats.

The systematic review and meta‐analysis of standardized Fist Few X cases and household transmission investigations for SARS‐CoV‐2 included data from 32 countries including Colombia, with 51% of them from 15 low‐ and middle‐income countries. The data obtained from this study were incorporated into the systematic review, contributing to the overall analysis of transmission patterns and epidemiological characteristics in diverse settings. This information is essential to support evidence‐based pandemic preparedness and response efforts not only for COVID‐19 but also for influenza and future novel respiratory viruses [11].

The epidemiological behavior of COVID‐19 does not differ from that reported in previous studies in terms of presentation stratified by age groups and clinical conditions; a similar distribution of cases was observed in both sexes, which contrasts with the series of the first 100 COVID‐19 cases from Lusaka, the capital of Zambia, in which the cases were predominantly male [12]. Children were least affected in the first decade of life, which is consistent with one of the largest data series published by the Chinese Centre for Disease Control and Prevention [13].

The highest percentage of confirmed cases was observed in the 25–55 age group; the age of the contacts was evenly distributed with a slightly higher percentage in those aged ≥ 65 years, which contrasts with other studies that reported clear differences in the age range of the contacts [14]. These variations may be influenced by demographic differences, expositions, household structures, and social and working interaction patterns in different settings. In some populations, older adults may have had higher exposure due to multigenerational living arrangements or caregiving roles, while in others, younger individuals might have had more frequent social or occupational exposures. Additionally, differences in testing strategies, case detection, and healthcare‐seeking behavior could also contribute to these discrepancies.

The highest percentage of people per household was in the range of 2–5 people and 1–2 people per room. These findings highlight the challenges of reducing transmission in the home environment, where residents cannot distance themselves from an infectious individual. This is in line with previous studies [15, 16], which reported that intra‐household dynamics are a risk factor for COVID‐19 transmission. The exposure of cases in open areas or at mass events was low, which is in line with other studies that reported low percentages of transmission in open areas (below 10% or 1%) [17, 18, 19].

Hospitalizations occurred in a relatively low percentage of cases, and complications due to SARS‐CoV‐2 were even lower; these findings were comparable to those in previous series reported in the early stages of the pandemic [14, 20, 21]. Intensive care admission occurred in 30% of the hospitalized cases, which is like the UK series (22.7%) [9].

The most common symptoms in the cases were like those reported in the literature [14, 21, 22]; fever, which was previously identified as one of the main tracer symptoms of infection [14, 23, 24] and had good sensitivity and specificity [7], was present in our series at a lower percentage than that reported in the literature.

The overall secondary clinical attack rate was 16.2%. Among all contacts, 31.4% developed clinical symptoms; however, half of these symptomatic individuals did not have laboratory confirmation of infection. This suggests that some cases may have gone undetected due to diagnostic limitations, or that their symptoms were caused by other conditions unrelated to the infection. This finding at the onset of new viral transmission is important for determining containment measures beyond diagnostic confirmation, a fact supported by our finding of a seroprevalence of 8%, which can be considered high for this early stage of the pandemic.

Moreover, 35.2% of the COVID‐19‐positive individuals confirmed by PCR, including both cases and contacts, were asymptomatic. Notably, the proportion of asymptomatic contacts, confirmed either by PCR or seroconversion, was high (58.4%), aligning with findings reported in the literature. This does not imply the absence of infection with this virus, as evidenced by the 6.1% of the contacts (22/360) who were asymptomatic and had positive PCR tests and positive IgG antibodies in the first samples, suggesting that they may even correspond to co‐primary cases. However, previous reports of symptoms in confirmed cases showed contrasting findings. In the study by Geteneh et al. [25], 85% of the patients were asymptomatic, whereas 18% of the patients were asymptomatic in a report from the quarantined Diamond Princess ship in Japan [26].

The time of appearance of IgG antibodies is not necessarily restricted to the commonly described period of 14 day [27], which may refer to the first appearance or another specific statistical point in the immune response; a group of individuals in the case series, who were positive for SARS‐CoV‐2, had detectable antibodies in the first collected sample, which may be explained either by early production of IgG antibodies or by the fact that the individual's first contact with the source of infection occurred long before the onset of symptoms. In addition, some laboratory results have shown that individuals with SARS‐CoV‐2 infection do not have detectable antibodies against SARS‐CoV‐2.

The presence of memory antibodies (IgG) is associated with disease severity. A study by Takeshita et al. [28] revealed a strong relationship between low Cq values reported by RT‐PCR, indicative of high viral load levels, and the antibody titer present in the studied individuals. These findings align with our results, where we analyzed the association between viral load and seroconversion (Figure 3). Specifically, we observed that patients with a high viral load had a greater proportion of seroconversion than those with a low relative viral load. This reinforces the notion that the magnitude of the initial viral exposure may influence the humoral immune response, as also suggested by Takeshita et al. [28].

The most important pre‐existing conditions were hypertension (prevalence, 24% in the Colombian population) [29] and diabetes. Both these conditions are among the top 10 causes of death in Colombia among both sexes [30]. Hypertension is reported to be among the most important pre‐existing health conditions of infected people [14, 23, 25]. Similarly, we reported that hypertension outweighed the other predominant health conditions, such as diabetes, asthma, and obesity.

The rate of secondary COVID‐19 attack in our study was 43%, which is very similar to that reported in the study by Hsu et al. [31] who estimated that 44% of the household contacts were infected with SARS‐CoV2. PCR‐RT of all close contacts, not just symptomatic contacts, provides the accurate rate of infectious attack, which was much higher than the clinical attack rate. In their contact tracing study, Kuba et al. [24] reported an attack rate of 12.1%, which included sampling of symptomatic household contacts only.

In a meta‐analysis conducted to evaluate the viral incubation periods, the mean incubation period was estimated to be in the range of 5.6–6.7 days, with the 95th percentile being 12.5 days, and it showed variations with age and sex [32]; the mean incubation period in the present study was 4.6 days with a median of 4 days, data that, although close to that of other studies, is smaller and perhaps more precise due to the rigor in monitoring the dates of exposure and onset of symptoms when this type of protocol is applied for the first few X cases.

The time interval between the onset of symptoms in primary and secondary cases (serial interval) was 4.7 days. Previous studies examining cohabitants and close contacts reported serial intervals of 3 [33], 3.8 [34], 5, and 6 [24] days after the onset of symptoms in the first case. Such findings suggest that infection occurs immediately after the onset of symptoms in the first case; thus, immediate isolation is necessary to prevent transmission between family members [24].

In this protocol type, the time interval between specimen collection and reporting is a key factor for the early identification of contacts. Moreover, the diagnostic test result was the trigger for initiating follow‐up; thus, it is important to obtain results within 24 h for the accurate calculation of the generation times and evolution of symptoms.

During the investigation period of cases and contacts, the transmission of the influenza virus in Colombia and the Americas was limited or nonexistent. In this study, screening for influenza A and B was conducted in all cases and contacts; however, no positive samples were detected. The absence of influenza cases aligns with regional and global reports indicating a significant reduction in influenza transmission during the COVID‐19 pandemic. This decline has been attributed to the widespread implementation of non‐pharmaceutical interventions, such as mask‐wearing, social distancing, mobility restrictions, and sample strategies like the sentinel testing approach. The findings reinforce the notion that pandemic control measures not only impact the transmission of SARS‐CoV‐2 but can also influence the circulation of other respiratory viruses, including influenza [35].

The implementation of the protocol for obtaining indicators requires technical and logistical efforts in many areas, which must be coordinated and simultaneous with those of the public health response teams in the areas that routinely conduct initial epidemiological field investigations to identify and manage new cases having a potential pandemic event. Given that this study was conducted 4 months after the onset of the COVID‐19 pandemic, the most important finding was knowing how to implement the FFX protocol as an initiative for global sero‐epidemiological standardization to combat the emergence of new pathogens with pandemic potential. This was achieved because the study findings allowed us to obtain standardized data that were shared for the global analysis of the situation. Accordingly, it is recommended to evaluate and adjust the complex operational aspects in the implementation of the protocol to reduce the response time and data generation.

This study faced several limitations that likely affected the validity and generalizability of its findings. The dual responsibilities of the surveillance personnel—managing both routine public health duties and the research protocol—placed a significant operational burden on field teams. This may have compromised the consistency of contact tracing, sample collection, and follow‐up procedures, potentially leading to incomplete case detection and delayed diagnoses. Additionally, the 4‐month delay in implementing the protocol meant that recruitment began during established community transmission, rather than during the initial outbreak phase. This may have introduced selection bias by overrepresenting symptomatic or severe cases while underestimating asymptomatic or mild infections, skewing secondary attack rate estimates. The limited training of field staff in standardized data collection, symptom classification, and sample handling likely contributed to inconsistencies in data quality across sites. These issues may have affected the accuracy of key epidemiological indicators such as incubation period, serial interval, and infection rates. While the study provided valuable insights into COVID‐19 transmission dynamics in a real‐world setting, these limitations should be considered when interpreting the results or applying them to broader contexts.

Based on the study's findings, several recommendations can be made to strengthen public health practice in the context of emerging infectious diseases. First, implementing protocols like FFX early in an outbreak is essential for capturing timely and representative data. Countries should integrate such protocols into national preparedness plans and ensure pre‐pandemic training of multidisciplinary field teams in protocol application, including standardized data collection, contact tracing, and biological sampling. Establishing rapid logistics and laboratory response systems is also critical to reduce delays in diagnosis and follow‐up.

Moreover, the high proportion of asymptomatic infections and seroconversion among contacts highlights the importance of combining molecular and serological testing in surveillance strategies to better estimate the true extent of transmission, especially in resource‐limited settings.

For future research, studies should aim to assess the performance of adapted FFX protocols when implemented earlier in an epidemic curve, ideally during the introduction phase of a novel pathogen. Additionally, further investigation is needed into the factors influencing variability in immune response—such as viral load, host characteristics, and comorbidities—which could improve risk stratification and guide targeted interventions. Comparative analyses of FFX implementations across different countries and settings would also help refine global standards for early outbreak investigations.

## Author Contributions

**Damaris Heredia:** project administration, investigation, formal analysis, writing – original draft preparation. **May Bibiana Osorio:** methodology, investigation, data curation, formal analysis, writing – original draft preparation. **Carolina Figueroa:** methodology, investigation, data curation, formal analysis, writing – original draft preparation. **Susanne Ardila:** methodology, investigation, data curation, formal analysis, writing – original draft preparation. **Dayner Vacca:** investigation, data curation, formal analysis, software. **Magdalena Santos:** investigation, collection, and analysis of case, collection, and analysis of survey and interview data, data curation. **Daniel Velandia:** investigation, analysis of case specimens. **Marcela Mercado:** investigation, analysis of case specimens, resources. **Maritza González:** project administration, resources, supervision, review and editing. **Franklyn Prieto:** resources, supervision, review and editing. **Ingrid Garcia:** conceptualization, project administration, review and editing. **Ángel Rodriguez:** conceptualization, review and editing. All authors reviewed the revisions for resubmission. The corresponding author prepared and wrote the responses to reviewers.

## Conflicts of Interest

The authors declare no conflicts of interest.

## Data Availability

The data are available upon reasonable request from the corresponding author.

## Endnotes

^1^
The definitions are found in the Instructions for intensified public health surveillance of acute respiratory infection and the disease associated with the new coronavirus 2019 (COVID 19). Version 13 of 31/12/2020 (INS, 2020). As the pandemic progressed, case definitions were adapted according to the event dynamics.
